# Antimicrobial and Antifungal Activity of Rare Substituted 1,2,3-Thiaselenazoles and Corresponding Matched Pair 1,2,3-Dithiazoles

**DOI:** 10.3390/antibiotics9070369

**Published:** 2020-07-01

**Authors:** Tuomo Laitinen, Ilia V. Baranovsky, Lidia S. Konstantinova, Antti Poso, Oleg A. Rakitin, Christopher R. M. Asquith

**Affiliations:** 1 School of Pharmacy, Faculty of Health Sciences, University of Eastern Finland, 70211 Kuopio, Finland; tuomo.laitinen@uef.fi (T.L.); antti.poso@uef.fi (A.P.); 2 N. D. Zelinsky Institute of Organic Chemistry, Russian Academy of Sciences, 119991 Moscow, Russia; ilay679@rambler.ru (I.V.B.); konstantinova_ls@mail.ru (L.S.K.); orakitin@ioc.ac.ru (O.A.R.); 3 Nanotechnology Education and Research Center, South Ural State University, Lenina Ave. 76, 454080 Chelyabinsk, Russia; 4 Department of Internal Medicine VIII, University Hospital Tübingen University of Tübingen, 72076 Tübingen, Germany; 5 Department of Pharmacology, School of Medicine, University of North Carolina at Chapel Hill, Chapel Hill, NC 27599, USA

**Keywords:** 1,2,3-dithiazole, 1,2,3-thiaselenazole, sulfur extrusion, selenium dioxide, antimicrobial, antibacterial, antifungal

## Abstract

We report our investigations into the underlying differences between 1,2,3-dithiazole and their ultra-rare counterpart, 1,2,3-thiaselenazole. This rare 1,2,3-thiaselenazole chemotype was afforded by sulfur extrusion and selenium insertion into the preconstructed 1,2,3-dithiazoles. We built a library of matched paired compounds to compare and contrast the two ring systems. This led to the development of both narrow and broad-spectrum antimicrobial compounds with sub-micro molar potency, limited to no toxicity and a further understanding of the transition state electronics through molecular simulations. We also identified the potent 4,5,6-trichlorocyclopenta[*d*][1,2,3]thiaselenazole **11a**, for use against *Candida albicans*, *Cryptococcus neoformans var. grubii*, *Staphylococcus aureus* and *Acinetobacter baumannii*, all of which have limited clinical treatment options. The 1,2,3-thiaselenazole represents a new class of potential compounds for the treatment of a host of multi-resistant hospital derived infections.

## 1. Introduction

Despite the advances in antibiotics and antifungals in recent years, there is an ever-increasing resistance emerging, with a real need for innovative solutions to reduce the infection burden [1]. Antimicrobial drugs can be divided into groups based on the mechanism of action. These inhibitors include agents targeting protein synthesis, nucleic acid synthesis, cell wall synthesis, metabolic pathways and drugs that cause depolarization of the cell membrane [2,3].

The development of novel antifungal drugs that do not have cross-resistance with current agents is an increasing challenge [4,5]. There are three major classes of antifungals currently used clinically for the treatment of invasive fungal infections [6]. This is compared to a plethora of options for bacterial infections [7]. These three classes include polyenes that sequester ergosterol which destabilizes the fungal cell membrane [8], and azoles that directly inhibit the biosynthesis of ergosterol, the major sterol in the fungal membrane [9]. The last class, which is a more recent addition to the clinical arsenal, echinocandins, inhibits synthesis of β-(1,3) glucan, which is a key component of the fungal cell wall [10,11]. Resistance is an acute clinical problem, with fungal infections having the same issues as multi-resistant bacteria infections, increasing the barrier to a successful cure [12].

**Figure 1:**
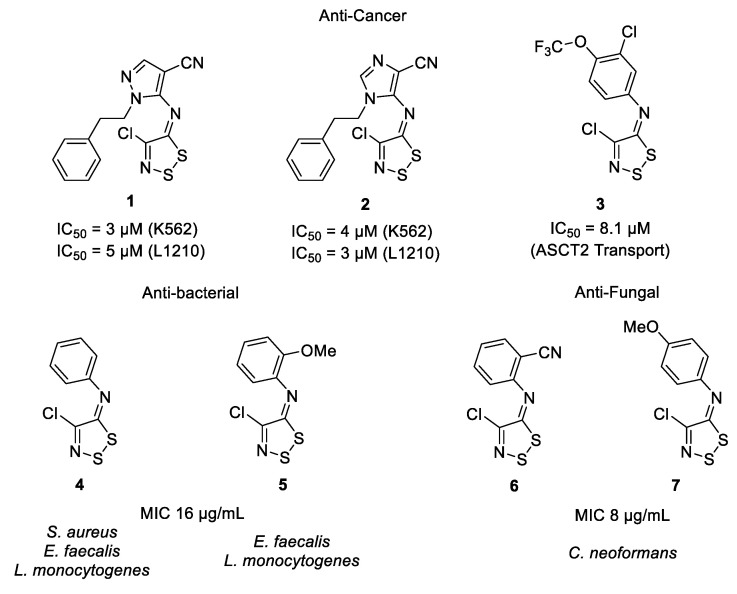
Examples of previously reported biologically active 5-imino-1,2,3-dithiazoles.

In our search for a solution to address the need for new antifungal and antibacterial agents we investigated the 1,2,3-thiaselenazole and 1,2,3-dithiazole scaffolds. The 1,2,3-dithiazole core has provided a number of interesting medicinal chemistry applications (Figure 1). In fact, 1,2,3-dithiazole scaffold has a broad biological activity profile which includes inhibition of cancer (**1**−**3**), [13,14,15] bacteria (**4**,**5**), [13,16,17,18,19] fungus (**6**,**7**) [19,20,21,22,23,24], virus [25,26] and melanin [27]. Although not definitively proven in most cases it is thought that most of these activities have centered around a cysteine-activated nucleophilic attack. With this mechanism in mind and previous success modulating that activity of 1,2,3-dithiazole, we also screened the corresponding 1,2,3-thiaselenazole analogs [13,25,26]. This was done to improve potency by weakening the S–S bond by elongation to more diffuse S–Se bond, while being careful to not compromise the toxicity profile of the compounds [26]. This, coupled with the knowledge that one of the three main compounds commercially used to suppress fungal growth is selenium sulfide, which is used for topical applications (used to treat skin and crops respectively) [28,29,30,31].

## 2. Results

### 2.1. Synthesis of 1,2,3-Dithiazoles and 1,2,3-Thiaselenazoles

**Scheme 1:**
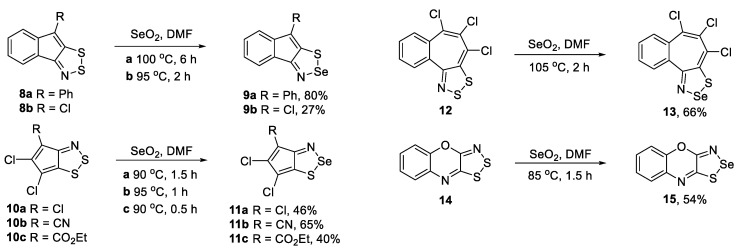
Synthesis route and conditions of tested analogs.

While the 1,2,3-dithiazoles synthesis is well described [32,33,34,35,36,37,38,39,40,41,42,43,44,45,46], 1,2,3-thiaselenazole presented a serious synthetic challenge with relatively few methods available for construction [26,47,48,49]. Two literature reports assisted in our route selection to access the selected 1,2,3-thiaselenazoles [26,47]. We utilize a sulfur extrusion method with a selenium insertion strategy starting from the corresponding 1,2,3-dithiazoles (Scheme 1). These 1,2,3-dithiazoles were created using functionalized oxime intermediates [25,26,50]. The 1,2,3-dithiazoles were then reacted with 10 equivalents of selenium dioxide in dimethylformamide (DMF) at temperatures between 80−110 °C. There is no product conversion observed at lower temperatures (<80 °C). However, selenium dioxide decomposes with prolonged heating in DMF at around 100 °C, so a careful substrate-specific optimization is essential. Despite reaction condition screening varying both temperature and solvent, excess selenium dioxide was always required to compensate for decomposition, which occurred under these relatively harsh conditions of DMF at about 100 °C [47].

To access compounds **8**−**15**, we first investigated the reaction between 8-phenylindeno[1,2-*d*]-1,2,3-dithiazole (**8a**) and selenium dioxide as this was the compound with the most benign substitution pattern. **8a** showed that no reaction occurred in most organic solvents including chloroform, benzene, THF, methanol, ethanol, 1,4-dioxane, acetonitrile, and DMSO. Treatment of 8-phenylindeno[1,2-*d*]-1,2,3-dithiazole (**8a**) with SeO_2_ in DMF led to successful exchange of sulfur to selenium in 1,2,3-dithiazole ring with the formation of thiaselenazole **9a** in high yield. The reaction was quite sensitive to temperature; no reaction occurred up to 90 °C and heating the reaction mixture above 110 °C led to violent decomposition due to the reaction of SeO_2_ with DMF. Under reduced temperature (95 °C), 8-chloroindeno[1,2-*d*]-1,2,3-dithiazole (**9b**) gave a much lower yield of 1,2,3-thiaselenazole (**9b**), an indicating that careful selection of the reaction temperature is needed in every case. A large excess of SeO_2_ (10 equiv.) was required for full conversion of dithiazole due it part to degradation of SeO_2_ in DMF in these harsh conditions. Treatment of other 1,2,3-dithiazoles (**10a-c**) under similar conditions furnished the corresponding 1,2,3-thiaselenazole (**11a-c**) in moderate to good yields, with highly tuned reaction conditions. The conversion **12** to **13** required a slightly higher temperature (105 °C), while the reaction of **14** to **15** was achieved at a much more modest temperature of 85 °C.

### 2.2. Evaluation of 1,2,3-Dithiazoles and 1,2,3-Thiaselenazoles

We then screened this series of 1,2,3-dithiazoles and 1,2,3-thiaselenazoles (**8**−**15**) against *Staphylococcus aureus*, *Acinetobacter baumannii*, *Candida albicans* and *Cryptococcus neoformans var. grubii* (Table 1) as part of the Community for Antimicrobial Drug Discovery (CO-ADD) initiative to develop new lead compounds [51]. These represent three distinct classes of microbes. *Staphylococcus aureus* (ATCC 43300) is a gram-positive bacterium derived from the super bug methicillin-resistant *Staphylococcus aureus* (MRSA) [52]. *Acinetobacter baumannii* (ATCC 19606) is a gram-negative bacterium hospital-derived infection with very few antibiotics that can treat this pathogen [53,54]. *Candida albicans* (ATCC 90028) and *Cryptococcus neoformans var. grubii* (ATCC 208821) are both opportunistic fungi [55,56,57,58]. These fungal pathogens are particularly dangerous for patients in hospital with immunodeficiencies, undergoing cancer chemotherapy and organ transplantation [59,60,61]. These strains are considered by the World Health Organization (WHO) to be the highest priority to develop novel antibiotics for control of these bacteria and fungi [62]. These are some of the most difficult to treat, particularly in a hospital setting where sepsis and pneumonia can quickly overcome the body’s immune system [62].

The compounds (**8**−**15**) were first screened and active (MIC ≤ 16 μg/mL or ≤ 10 μM) compounds progressed to full dose response, toxicity studies in HEK293 cells and a haemolysis assay. 4,5,6-Trichlorocyclopenta[*d*][1,2,3]dithiazole (**10a**) was inactive in the initial screen. However, the corresponding 1,2,3-thiaselenazole (**11a**) was very potent across all classes of pathogen with no haemolysis and mild toxicity (CC_50_ = 0.52 μM). The introduction of a nitrile substituent to the 4-postion of the dithiazole (**10b**) removed all activity against *A. baumannii* and *S. aureus* but maintained activity against the fungi with no toxicity. The matched pair 1,2,3-thiaselenazole (**11b**) had a similar profile but was also potent against *S. aureus*. Exchanging the nitrile for an ethyl ester (**10c**) made the compound selective for C. *albicans* with no toxicity. The 1,2,3-thiaselenazole (**11c**) had a much broader potent activity against three of the four pathogens with only limited toxicity (CC_50_ = 7 μM) and no haemolysis. The introduction of an additional integrated phenyl to form a 7-membered ring 4,5,6-trichlorobenzo[6,7]cyclohepta[1,2-*d*][1,2,3]dithiazole (**12**) which was inactive. The 1,2,3-thiaselenazole analog (**13**) was potent on against three of the four pathogens with a similar profile to **9a** without the activity on *A. baumannii*. The compound with the substitution to form 8-chloroindeno[1,2-*d*][1,2,3]dithiazole (**8a**) was inactive, but the corresponding 1,2,3-thiaselenazole (**8a**) was antifungal selective as with **10b** and showed no toxicity or haemolysis. The further substitution of the final chlorine was inactive both as the 1,2,3-dithiazole (**8b**) and 1,2,3-thiaselenazole (**9b**). A change in the electronegativity profile to form benzo[*b*][1,2,3]dithiazolo[5,4-*e*][1,4]oxazine (**14**) was not fruitful, but the matched pair 1,2,3-thiaselenazole (**15**) recovered some antifungal activity with no toxicity. The compounds (**8**−**15**) were also screened against *E. coli*, *K. pneumoniae*, *P. aeruginosa* and were all inactive (MIC ≤ 16 μg/mL). 

### 2.3. Modelling Investigation

The nature of the proposed nucleophilic mechanism means that the sulfur–sulfur or sulfur–selenium bond is central to the activity profile of the compounds. We were interested to see if the incorporation of selenium into this system to perturb the electron density could enhance the activity profile. We modelled and synthesized a series of matched pairs to investigate these effects. We modelled the Fukui indices as previously described on the system [26]. Then, we investigated the vibrational energies of each ring system along with the electron density profiles and the reaction coordinates.

**Figure 2:**
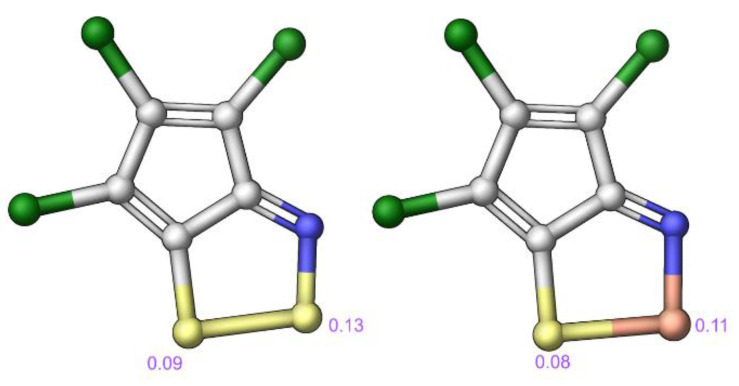
Fukui f-NN-LUMO indices for **10a** (model for 1,2,3-dithiazoles) vs **11a** (model for 1,2,3-thiaselenazoles). The atoms are colored: yellow—sulfur; bronze—selenium; blue—nitrogen; grey -carbon; green—chlorine.

**Figure 3:**
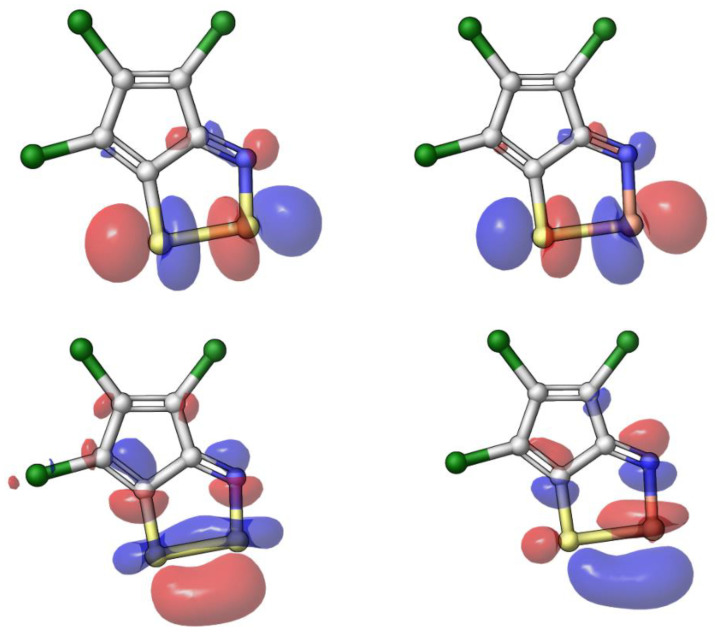
Upper row shows LUMO+1, which suggest directions of the potential scissile bond. Lower row shows empty LUMO+2 orbital, which are thought to be available for nucleophilic attack by negatively charged highly nucleophilic cysteine. The atoms are colored: yellow—sulfur; bronze—selenium; blue—nitrogen; grey—carbon; green—chlorine.

Atomic Fukui indices suggest that both 1,2,3-dithiazole and 1,2,3-thiaselenazole ring systems are susceptible to nucleophilic attack of an assumed negatively charged cysteine (Figure 2). If the scaffold is behaving as a ligand/substrate, the atoms that are most reactive towards electrophilic attack are indicated by high positive values of f−NN for the HOMO. This hypothesis is further supported by location of LUMO orbitals (Figure 3). The LUMO+1 might show bond cleavage and LUMO+2 orbital is showing location of potential empty orbital that is available for a nucleophilic attack (Figure 3).

**Figure 4:**
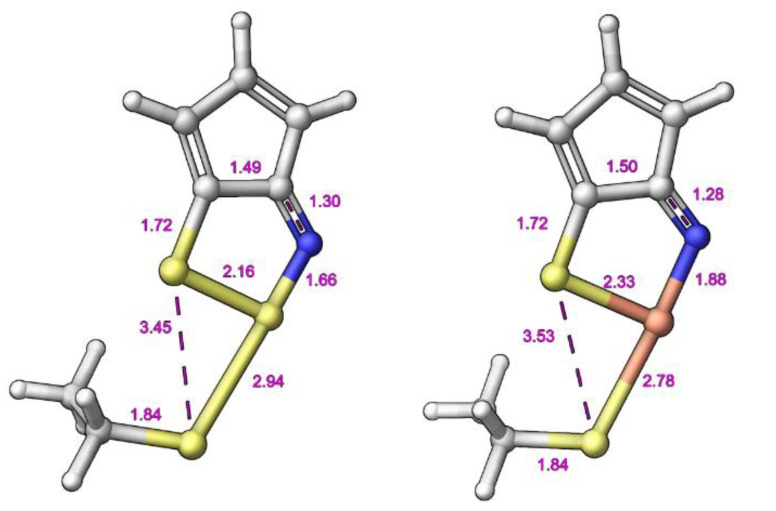
Transition state modelling of 1,2,3-dithiazole vs 1,2,3-thiaselenazole ring opening upon nucleophilic attack in model system. Numbers in purple show distance in Å. The selenium compound is missing the pseudo grids caused by convergence problems and cc-PVTZ(-F) basis set was used for transition states calculations in Jaguar.

**Figure 5:**
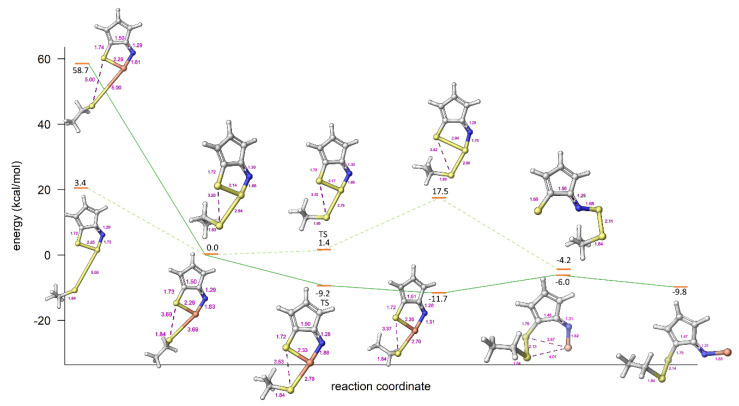
Advanced Transition State Modelling Investigation: Estimated relative reaction energy diagram for the ring opening calculated point wise using model compounds and B3LYP/cc-PVTZ(-F) level of theory. The dashed line shows the relative energetics for 1,2,3-dithiazoles, **10a** type compounds and continuous green line shows reaction path for corresponding 1,2,3-thiaselenazoles, **11a** selenium derivatives. Stages of the reaction are from left to right: isolated reactant complex, reactant complex, transition state (merged as TS) and theoretical products. Selected bond distances are shown using numbers in magenta color.

The vibrational analysis of **10a** and **11a**, demonstrates that only 1,2,3-thiaselenazole (**11a**) shows negative vibrations located exactly in the selenium center of the 1,2,3-thiaselenazole ring system (Table 2). This is thought to be one indication for a potentially reactive system. When transition state optimization was conducted, the 1,2,3-thiaselenazole model showed later transition state geometry and suggested a probable ring opening via nucleophilic attack (Figure 4). The proposed full reaction profile is now reported in detail (Figure 5). Some points were calculated with distant constraints or as single point energies due to convergence problems at the reactant stage of the reaction path. Structures in the reaction energetic diagram are fully gas phase optimized (B2LYP/cc-PVTZ(-F), except isolated reactants and the reactant complex of the **11a** model compound [63]. The energy for isolated reactants was calculated using distant constraint between negatively charged reactant cysteine and ligand. The energy estimation for the reactant complex of model compound **11a** was calculated using geometry taken from a partly optimized structure. The final optimization was calculated by constraining the reactive distance to a normalized value (3.69Å). The further optimization of this reactive species resulted in a downward trajectory towards an energetically more favorable product. Transition state structures were further validated by following the reaction path downhill using IRC functionality of Jaguar.

## 3. Discussion

Hospital-acquired infections are an issue worldwide adding to the already strained resources of health services and striking at the most vulnerable in society [62,64,65]. The Community for Antimicrobial Drug Discovery (CO-ADD) initiative provides a mechanism to generate new lead compounds to address these multi-resistant and opportunistic infections [51].

We have successfully identified compounds with broad activity and limited toxicity that were previously unknown, highlighting the value of this approach. The compound 4,5,6-trichlorocyclopenta[*d*][1,2,3]thiaselenazole (**11a**) stands out as a compound that is active against *Candida albicans*, *Cryptococcus neoformans var. grubii*, *Staphylococcus aureus* and *Acinetobacter baumannii*, all of which have limited clinical treatment options. On the same scaffold we were able to modulate activity across all four pathogens without significant effects on the toxicity profile, all of which was supported by extensive modelling efforts. The other compounds tested within this series add weight to the idea that this series is tractable for further development.

This rare 1,2,3-thiaselenazole chemotype along with the more common 1,2,3-dithiazole have been underrepresented in the medicinal chemistry literature for many years despite encouraging biological profiles [13,14,15,16,17,18,19,20,21,22,23,24,25,26,27]. Despite the unconventional structure we were able to construct a mechanistic rational based on the activity profile between the matched pairs through molecular simulations and quantum mechanics. This highlighted that the sulfur–sulfur vs sulfur–selenium bond strength was key for lowering the transition state energy with limited impact on non-specific toxicity.

This is only the second biological evaluation of a 1,2,3-thiaselenazole scaffold. This initial series of results is an exciting first step in defining a medicinal chemistry trajectory towards a series of optimized antimicrobial and antifungal compounds with potential to treat multi-resistant bacteria and fungal infections.

## 4. Materials and Methods

### 4.1. Chemistry

#### 4.1.1. General Procedure for Synthesis of 1,2,3-dithiazoles.

S_2_Cl_2_ (0.24 mL, 3.0 mmol; or 0.48 mL, 6.0 mmol) was added dropwise to a stirred solution of cyclic oxime (1.0 mmol) [25,66] and pyridine (0.32 mL, 4.0 mmol; or 0.64 mL, 8.0 mmol) in dry MeCN (10 mL) at −25 °C and under argon. The mixture was stirred for 0.5 h at −5–0 °C, then for 24 h at ambient temperature, followed by a 1 h reflux, filtering and then the solvent was distilled off under reduced pressure. The residue was dissolved in EtOH (20 mL), diluted by H_2_O (20 mL) and extracted with ether (3 × 20 mL). Combined extracts were washed with H_2_O (20 mL), dried, and the solvent was evaporated [26,47].

8-Phenylindeno[1,2-*d*]-1,2,3-dithiazole (**8a**). Yellow solid, mp 111–112 °С (111–113 °С). IR, MS and NMR spectra are similar to the literature data [67].

8-Chloroindeno[1,2-*d*]-1,2,3-dithiazole (**8b**). Red solid, mp 108–110 °С (107–109 °С). IR, MS and NMR spectra are similar to the literature data [67].

4,5,6-Trichlorocyclopenta[*d*][1,2,3]dithiazole (**10a**). Deep purple solid, mp 122–124 °С (125–127 °С). IR, MS and NMR spectra are similar to the literature data [67].

5,6-Dichlorocyclopenta[1,2,3]dithiazole-4-carbonitrile (**10b**)**.** Deep purple solid, mp 217–219 °С (217–218 °С). IR, MS and NMR spectra are similar to the literature data [68].

Ethyl 5,6-dichlorocyclopenta[*d*][1,2,3]dithiazole-4-carboxylate (**10c**). Yellow solid, mp 80–82 °С (83–84 °С). IR, MS and NMR spectra are similar to the literature data [69].

4,5,6-Trichlorobenzo[6,7]cyclohepta[1,2-*d*][1,2,3]dithiazole (**12**). Red solid, mp 119–121 °С (121–122 °С). IR, MS and NMR spectra are similar to the literature data [67].

Benzo[*b*][1,2,3]dithiazolo[5,4-*e*][1,4]oxazine (**14**). Red solid, mp 182–184 °С (183–184 °С). IR, MS and NMR spectra are similar to the literature data [70].

#### 4.1.2. General Procedure for Synthesis of 1,2,3-Thiaselenazoles

Under argon, a stirred mixture of 1,2,3-dithiazole (1.00 mmol) and SeO_2_ (1.11g, 10.0 mmol) in dry DMF (7 mL) was heated for the time and at the temperature specified in Scheme 1. The reaction mixture was cooled to 20 °C and filtered. The solid was washed with CH_2_Cl_2_ (3 mL) and 100 mL of CH_2_Cl_2_ was added to the filtrate. The combined liquors were washed with brine (3 × 30 mL) and dried over MgSO_4_. The solvent was distilled off under reduced pressure, and the residue was purified by silica column chromatography (Merck 60, hexane / CH_2_Cl_2_ mixtures) [47].

8-Chloroindeno[1,2-*d*][1,2,3]thiaselenazole (**9a**). Black crystals, yield 27%, mp 142–144 °C. Anal. calcd. for С_9_H_4_ClNSSe (272.61): С, 39.65; H, 1.48; N, 5.14; Se, 28.96. Found: C, 39.33; H, 1.32; N, 5.32; Se, 29.25. ESI-MS: found m/z 272.8914; calcd. for С_9_Н_4_^35^ClNS^78^Se [M]^+^ 272.8909. MS, m/z (%): 275 ([M]^+^, 27), 273 (M^+^, 63), 271 (M^+^, 29), 195 (62), 193 (61), 158 (50), 114 (100). NMR, δ: ^1^H (CDCl_3_): 7.89 (1H, d, CH, ^3^J = 7.3 Hz), 7.49 (1H, m, CH), 7.33 (2H, m, 2CH); ^13^C (DMSO-d_6_): 139.5, 127.5 (CH), 126.9, 125.5 (CH), 124.8 (CH), 124.7, 122.0, 114.9 (CH). IR (KBr), ν, cm^–1^: 2957, 2851, 1601, 1407, 1252, 1093, 752.

8-Phenylindeno[1,2-*d*][1,2,3]thiaselenazole (**9b**). Black crystals, yield 80%, mp 195–197 °C. Anal. calcd. for С_15_H_9_NSSe (314.26): С, 57.33; H, 2.89; N, 4.46; Se, 25.13. Found: C, 57.02; H, 2.75; N, 4.62; Se, 25.34. ESI-MS: found m/z 314.9624; calcd. for С_15_Н_9_NS^78^Se [M]^+^ 314.9615. MS, m/z (%): 315 ([M]^+^, ^80^Se; 52), 235 (50), 203 (100). NMR, δ: ^1^H (CD_2_Cl_2_): 7.99 (1H, d, 2CH, ^3^J = 7.4 Hz), 7.68 (2H, m, CH), 7.61 (3H, m, CH), 7.46 (2H, m, CH), 7.35 (1H, m, CH); ^13^C (DMSO-d_6_): 150.0, 135.3, 131.0, 129.8, 129.7, 129.2 (C-H), 128.6 (CH), 127.5 (CH), 127.4 (CH), 127.0 (CH), 124.9, 123.7 (CH), 120.0 (CH); ^77^Se (DMSO-d_6_): 1435. IR (KBr), ν, cm^–1^: 3050, 2923, 1600, 1577, 1453, 1339, 1294, 1281, 1121, 767, 690.

4,5,6-Trichlorocyclopenta[*d*][1,2,3]thiaselenazole (**11a**). Black crystals, yield 46%, mp 117–119 °C. Anal. calcd. for С_5_Cl_3_NSSe (291.44): С, 20.61; N, 4.81; Se, 27.09. Found: C, 20.42; N, 5.03; Se, 27.52. MS, m/z (%): 291 ([M]^+^, 20), 256 ([M–Cl] ^+^, 36), 211 (9), 176 (5), 64 (100). NMR, δ: ^13^C NMR (DMSO-d_6_): 144.6, 123.4, 120.3, 120.1, 119.9. IR (KBr), ν, cm^–1^: 1562, 1542, 1263, 1204, 1087, 885, 775.

5,6-Dichlorocyclopenta[*d*][1,2,3]thiaselenazole-4-carbonitrile (**11b**). Black crystals, yield 65%, mp 171–173 °C. Anal. calcd. for С_6_Cl_2_N_2_SSe (282.01): С, 25.55; N, 9.93; Se, 28.00. Found: C, 25.35; N, 10.12; Se, 28.34. ESI-MS: found m/z 282.8391; calcd. for С_6_Cl_2_N_2_S^78^Se [M+H]^+^ 282.8391. MS, m/z (%): 282 ([M]^+^, ^80^Se; 20), 202 (12), 167 (6), 132 (20). NMR, δ: ^13^C (DMSO-d_6_): 170.0, 154.1, 130.6, 130.0, 126.5, 80.4. IR (KBr), ν, cm^–1^: 2223, 1629, 1442, 1317, 1242, 1097, 1028, 802.

Ethyl 5,6-dichlorocyclopenta[*d*][1,2,3]thiaselenazole-4-carboxylate (**11c**). Black crystals, yield 40%, mp 188-190 °C. Anal. calcd. for С_8_H_5_Cl_2_NO_2_SSe (329.06): С, 29.20; H, 1.53; N, 4.26; Se, 24.00. Found: C, 29.42; H, 1.65; N, 4.08; Se, 24.32. ESI-MS: found m/z 351.8458; calcd. for С_8_H_5_Cl_2_NO_2_S^78^Se [M+ Na]^+^ 351.8470. MS, m/z (%): 329 ([M]^+^, 25), 284 (50), 257 (50). NMR, δ: ^1^H (CDCl_3_) δ: 4.43 (2H, q, CH, J = 7.3 Hz), 1.42 (3H, t, J = 7.3 Hz, CH_3_); ^13^C (CDCl_3_): 166.3, 160.7, 156.6, 141.8, 114.8, 109.9, 60.8, 14.4. IR (KBr), ν, cm^–1^: 2853, 2924, 1668, 1443, 1313, 1270, 1190, 1095, 654.

4,5,6-Trichlorobenzo[6,7]cyclohepta[1,2-*d*][1,2,3]thiaselenazole (**13**). Black crystals, yield 66%, mp 144–145 °C. Anal. calcd. for С_11_H_4_Cl_3_NSSe (367.54): С, 35.95; H, 1.10; N, 3.81; Se, 21.48. Found: C, 35.74; H, 1.02; N, 4.09; Se, 21.67. ESI-MS: found m/z 367.8353; calcd. for С_11_H_4_Cl_3_NS^78^Se [M+H]^+^ 367.8360. MS, m/z (%): 367 ([M]^+^, 100), 332 (40), 287 (39), 255 (39), 217 (80), 182 (56), 138 (54). NMR, δ: ^1^H (CD_2_Cl_2_): 7.90 (1H, d, CH, ^3^J = 7.4 Hz), 7.49 (1H, m, CH), 7.33 (1H, d, CH, ^3^J = 7.9 Hz); ^13^C (CD_2_Cl_2_): 161.4, 154.6, 137.5, 134.2, 133.3, 132.8, 132.4 (CH), 132.0 (CH), 131.3 (CH), 131.4 (CH), 131.1. IR (KBr), ν, cm^–1^: 2922, 2852, 1541, 1506, 1148, 1087, 750, 669.

Benzo[*b*][1,2,3]thiaselenazolo[5,4-*e*][1,4]oxazine (**15**). Orange crystals, yield 54%, mp 178–180 °C. Anal. calcd. for С_8_H_4_N_2_OSSe (255.16): С, 37.66; H, 1.58; N, 10.98; S, 12.57; Se, 30.95. Found: C, 37.71; H, 1.65; N, 10.86; S, 12.76; Se, 31.18. ESI-MS: found m/z 256.9282; calcd. for С_8_H_4_N_2_OS^78^Se [M+H]^+^ 256.9282. MS, m/z (%): 256 ([M]^+^, ^80^Se; 77), 208 (2), 192 (8), 167 (30). NMR, δ: ^1^H (DMSO-d_6_): 7.53 (2H, m, CH), 7.31 (2H, m, CH); ^13^C (DMSO-d_6_): 173.1, 155.1, 132.1 (CH), 131.4, 131.0 (CH), 130.4, 128.9, 126.4 (CH), 125.8 (CH), 120.7; ^77^Se (DMSO-d_6_): 1012. IR (KBr), ν, cm^–1^: 1562, 1474, 1457, 1280, 1233, 1043, 915, 753.

### 4.2. Molecular Simulations and Modelling

#### 4.2.1. General Modelling and Electronic Structure Calculations

Schrödinger Maestro was used for all modelling tasks. Small molecules were first prepared using Ligprep module. Initial guesses for model compounds were constructed and structure manipulations as well as visualization of the results were done with Maestro GUI. Electronic structure calculations were computed using Jaguar module of Schrödinger suite. (Schrödinger Release 2020-1: Jaguar, Schrödinger, LLC, New York, NY, USA, 2020). Initial gas phase optimization for compounds were done using density functional theory B3LYP and basis set of 6-31G**. Thus, the most interesting ligands contained fourth row selenium atoms and for those, pseudo spectral grids are only available on higher basis sets. As a consequence, all reported geometry optimizations were finally carried out using B3LYP/cc-PVTZ(-F), available in the Jaguar suite. Fukui indices and visualization of molecular orbitals were calculated for B3LYP/cc-PVTZ(-F) optimized gas phase geometries.

#### 4.2.2. Vibrational Analysis

Vibrational analysis was carried out for gas phase optimized B3LYP/cc-PVTZ(-F) geometries using corresponding theory and basis set.

#### 4.2.3. Transition State Optimization

The initial transition state calculation of ring opening of 1,2,3-dithiazoles and 1,2,3-thiaselenazoles upon negative cysteine nucleophilic attack is based on LUMO+2 orbitals from **10a** and **11a** models. Initial transition state optimization was calculated using a 1,2,3-dithiazole model system using B3LYP/6-31G** level of theory and basis set.

### 4.3. Biology

Antimicrobial screening was performed by CO-ADD (The Community for Antimicrobial Drug Discovery), Australia [51].

#### 4.3.1. Antibacterial Assay

All bacteria, *S. aureus* (Strain ATCC 43300, MRSA), *E. coli* (Strain ATCC 25922, FDA control strain), *K. pneumoniae* (Strain ATCC 700603, MDR), *A. baumannii* (Strain ATCC 19606, Type strain), *P. aeruginosa* (Strain ATCC 27853, Quality control strain), were cultured in Cation-adjusted Mueller Hinton broth (CAMHB) at 37 °C overnight. A sample of each culture was then diluted 40-fold in fresh broth and incubated at 37 °C for 1.5–3 h. The resultant mid-log phase cultures were diluted (CFU/mL measured by OD600), then added to each well of the compound containing plates, giving a cell density of 5 × 10^5^ CFU/mL and a total volume of 50 μL. All the plates were covered and incubated at 37 °C for 18 h without shaking.

Analysis: Inhibition of bacterial growth was determined measuring absorbance at 600 nm (OD600), using a Tecan M1000 Pro monochromator plate reader. The percentage of growth inhibition was calculated for each well, using the negative control (media only) and positive control (bacteria without inhibitors) on the same plate as references. The percentage of growth inhibition was calculated for each well, using the negative control (media only) and positive control (bacteria without inhibitors) on the same plate. The MIC was determined as the lowest concentration at which the growth was fully inhibited, defined by an inhibition ≥80%.

#### 4.3.2. Antifungal Assay

Fungi strains *C. albicans* (Strain ATCC 90028, CLSI reference) and *C. neoformans* (Strain ATCC 208821, H99 Type strain), were cultured for 3 days on Yeast Extract-Peptone Dextrose (YPD) agar at 30 °C. A yeast suspension of 1 × 10^6^ to 5 × 10^6^ CFU/mL (as determined by OD530) was prepared from five colonies. The suspension was subsequently diluted and added to each well of the compound-containing plates giving a final cell density of fungi suspension of 2.5 × 10^3^ CFU/mL and a total volume of 50 μL. All plates were covered and incubated at 35 °C for 36 h without shaking.

Analysis: Growth inhibition of *C. albicans* was determined measuring absorbance at 630 nm (OD630), while the growth inhibition of *C. neoformans* was determined measuring the difference in absorbance between 600 and 570 nm (OD600–570), after the addition of resazurin (0.001% final concentration) and incubation at 35 °C for 2 h. The absorbance was measured using a Biotek Multiflo Synergy HTX plate reader. In both cases, the percentage of growth inhibition was calculated for each well, using the negative control (media only) and positive control (fungi without inhibitors) on the same plate. The MIC was determined as the lowest concentration at which the growth was fully inhibited, defined by an inhibition ≥80% for *C. albicans* and an inhibition ≥70% for *C. neoformans*. Due to a higher variance in growth and inhibition, a lower threshold was applied to the data for *C. neoformans*.

#### 4.3.3. Cytotoxicity Assay

HEK293 cells were counted manually in a Neubauer haemocytometer and then plated in the 384-well plates containing the compounds to give a density of 6000 cells/well in a final volume of 50 μL. DMEM supplemented with 10% FBS was used as growth media and the cells were incubated together with the compounds for 20 h at 37 °C in 5% CO_2_. Analysis: Cytotoxicity (or cell viability) was measured by fluorescence, ex: 560/10 nm, em: 590/10 nm (F560/590), after addition of 5 μL of 25 μgmL^−1^ Resazurin (2.3 μgmL^−1^ final concentration) and after incubation for further 3h at 37 °C in 5% CO_2_. The fluorescence intensity was measured using a Tecan M1000 Pro monochromator plate reader, using automatic gain calculation CC_50_ (concentration at 50% cytotoxicity) were calculated by curve fitting the inhibition values vs. log(concentration) using sigmoidal dose-response function, with variable fitting values for bottom, top and slope.

#### 4.3.4. Haemolysis Assay

Hc10 (concentration at 10% haemolytic activity) was calculated by curve fitting the inhibition values vs. log(concentration) using a Sigmoidal dose-response function, with variable values for bottom, top and slope. The curve fitting is implemented using Pipeline Pilot’s dose-response component (giving similar results to similar tools such as GraphPad’s Prism and IDBS’s XlFit). The curve fitting resulted in Hc10 (50%) values, which are converted into Hc10 by Hc10 = Hc10*(10/90) ^ (1/Slope); any value with > indicates a sample with no activity (low D_Max_ value) or samples with Hc10 values above the maximum tested concentration (higher D_Max_ value). D_Max_ represents the highest percentage inhibition response for all concentrations tested. The value helps to indicate if samples are displaying only partial response at the screened concentration, suggesting that the sample might be fully active at a higher concentration or if the sample only exhibits partial inhibition. In addition, the value indicates if samples have been active, but curve fitting failed, mostly because only the single highest concentration was active.

## 5. Conclusions

The rare 1,2,3-thiaselenazole chemotype has showed exciting potential with sub-micro molar potency on a series of multi-resistant pathogens with limited to no toxicity. The identification of the potent 4,5,6-trichlorocyclopenta[*d*][1,2,3]thiaselenazole **11a** lead compound, for use against *Candida albicans*, *Cryptococcus neoformans var. grubii*, *Staphylococcus aureus* and *Acinetobacter baumannii* is a significant step forward. These pathogens all have limited clinical treatment options and are in desperate need of new therapies with novel mechanisms of action. This initial series of results, along with the identification of **11a** provides the foundation to defining a medicinal chemistry trajectory towards optimized inhibitors with potential to treat multi-resistant bacteria and fungal infections.

## Figures and Tables

**Table 1:** Antibacterial and antifungal activity of **8**−**15**.

Compound		X	Initial Screen ^a^	Antibacterial		Antifungal		CC_50_^b^	Hc10 ^c^
				*A. baumannii*	*S. aureus*	*C. albicans*	*C. neoformans*		
				MIC (μg mL^−1^)		MIC (μg mL^−1^)			
**10a**	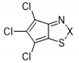	**S**	Inactive	-	-	-	-	-	-
**11a**		**Se**	Active	≤0.25	≤0.25	≤0.25	≤0.25	0.52	>32
**10b**	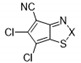	**S**	Active	>32	>32	≤0.25	≤0.25	>32	>32
**11b**		**Se**	Active	>32	≤0.25	≤0.25	≤0.25	>32	>32
**10c**	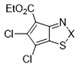	**S**	Active	>32	>32	≤0.25	>32	>32	>32
**11c**		**Se**	Active	>32	≤0.25	≤0.25	≤0.25	7	>32
**12**	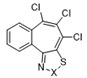	**S**	Inactive	-	-	-	-	-	-
**13**		**Se**	Active	>32	≤0.25	≤0.25	≤0.25	0.48	>32
**8a**	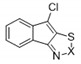	**S**	Inactive	-	-	-	-	-	-
**9a**		**Se**	Active	>32	>32	≤0.25	≤0.25	>32	>32
**8b**	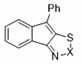	**S**	Inactive	-	-	-	-	-	-
**9b**		**Se**	Inactive	-	-	-	-	-	-
**14**	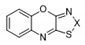	**S**	Inactive	-	-	-	-	-	-
**15**		**Se**	Active	>32	>32	≤0.25	2	>32	>32

^a^ Active was defined at >50% inhibition at 10 μM, MIC was determined as the lowest concentration at which the growth was fully inhibited, defined by an inhibition ≥ 80%; ^b^ Concentration at 50% cytotoxicity; ^c^ Concentration at 10% haemolytic activity.

**Table 2:** Vibrational frequencies calculated for gas phase optimized structures of compounds **10a** and **11a** calculated with B3LYP//cc-PVDZ(-F). Negative frequencies in the case of compound **11a** suggest reactivity of the selenium center.

Vibration	10a		Vibration	11a	
	Frequencies	Intensities		Frequencies	Intensities
1	81.19	0.090	1	−1106.90	1.59
2	97.19	0.050	2	−428.76	4.23
3	126.77	0.53	3	85.37	0.070
4	159.42	0.33	4	121.91	0.49
5	168.71	0.050	5	174.60	0.22
6	189.77	1.02	6	197.10	0.27
7	238.27	0.27	7	209.31	0.56
8	326.79	0.55	8	297.64	0.40
9	351.68	1.23	9	306.99	2.03
10	372.54	2.73	10	354.64	2.95
11	397.68	7.85	11	382.75	0.40
12	426.85	1.18	12	421.39	0.91
13	440.19	7.82	13	531.84	0.56
14	560.53	3.48	14	587.19	69.88
15	644.34	0.13	15	642.56	0.12
16	656.69	11.16	16	670.91	37.5
17	708.68	3.56	17	712.86	3.37
18	718.86	86.30	18	772.86	28.48
19	796.88	81.17	19	816.30	1.46
20	881.86	24.82	20	857.16	27.67
21	1007.65	0.60	21	997.94	1.43
22	1100.15	71.57	22	1092.41	70.45
23	1205.19	147.80	23	1201.72	177.49
24	1283.36	123.21	24	1271.67	108.05
25	1493.57	5.90	25	1513.89	7.19
26	1581.56	102.24	26	1584.26	94.69
27	1596.95	28.40	27	1609.23	33.50
